# Postnatal Rapid Weight Gain of Japanese Infants Based on the World Health Organization Child Growth Standards

**DOI:** 10.7759/cureus.97098

**Published:** 2025-11-17

**Authors:** Izumi Akaboshi

**Affiliations:** 1 Pediatric Endocrinology, Kumamoto City Medical Association/Akaboshi Pediatric Clinic, Kumamoto, JPN

**Keywords:** national reference, postnatal rapid weight gain, standard deviation score, weight-for-age, who z-score

## Abstract

There is limited research directly comparing WHO weight-for-age z-scores with the Japanese national reference values for infants. Furthermore, few studies have examined the prevalence of rapid weight gain from birth to at least three months of age using the standard deviation scores based on the national reference. However, no studies have been reported that investigate postnatal rapid weight gain in Japanese infants and preschool children using the WHO weight z-score. This study aims to convert the national references to WHO weight-for-age z-scores, to assess whether WHO z-scores at birth remain low by 2023 for both sexes, to examine trends in these weight growth patterns up to 2023, and to estimate the prevalence of postnatal rapid weight gain using the WHO weight-for-age z-scores and to evaluate whether this prevalence of postnatal rapid weight gain differs between WHO weight-for-age z-scores and standard deviation scores based on the national weight references. The database in this study used the community-based and cross-sectional national surveys, which comprised 14,115 Japanese infants and preschool children aged 14 days to six years at the time of measurement in 2000, 12,426 in 2010, and 11,194 in 2023. WHO weight-for-age z-scores were calculated using an automated conversion tool based on the Japanese national reference. Postnatal rapid weight gain was defined as a sex-specific increase in weight-for-age z-score or SD score of > 0.67. Across all percentiles in the years 2000, 2010, and 2023, the WHO weight-for-age z-scores remained - 0.6 or less at birth, peaked at one and a half months of age, and gradually declined thereafter. Weight-for-age z-score trajectories for both sexes were the highest in 2000 and showed a steady decline by 2023. The WHO weight-for-age z-scores produced a higher prevalence of postnatal rapid weight gain compared with the SD scores of Japanese references. Weight growth patterns based on WHO z-scores showed a steady downward trend through 2023. The prevalence of postnatal rapid weight gain assessed using WHO z-scores appears to be potentially overestimated. Caution is advised when evaluating research that uses different criteria for postnatal rapid weight gain.

## Introduction

In 2006, the World Health Organization Child Growth Standards (WHOCGS) were established to assess the growth and development of infants and children up to five years of age [1]. The WHOCGS were developed based on growth data collected from approximately 8,500 children across six countries - Brazil, India, Ghana, Norway, Oman, and the United States - representing a diverse range of ethnic backgrounds. All infants included in the study were exclusively breastfed for the first four months of life [1].

In Japan, a nationwide survey on the growth and development of infants and children has been conducted since 1960. The Ministry of Health, Labour and Welfare (MHLW) published national growth data for infants and preschool children in 2000, 2010, and the Children and Families Agency (CFA) released updated data in 2023. CFA is a governmental body in Japan, established in April 2023 as an external bureau of the Cabinet Office. Its purpose is to consolidate administrative functions related to children, which were previously divided between the Cabinet Office and the MHLW. These reports include sex- and age-specific percentile values for weight, adjusted using the LMS method. However, for the 2023 data, monthly percentile values for infants have only been reported up to 11.5 months of age, with subsequent data presented as six-month averages [2-4]. A previous study published in 2022 compared the WHO weight-for-age (WFA) z-scores with the Japanese national percentile references from 2000, but similar comparisons for the 2010 and 2023 references have not been conducted [5]. Thus, longitudinal analysis of weight growth trends across all three Japanese references is currently lacking.

The decline in mean birth weight (kg) and the increase in the prevalence of low birth weight infants (<2,500 g) in Japan have persisted consistently since 1980 and have not been fully corrected as of 2023.

Postnatal rapid weight gain (PRWG) within the first two years of life is considered a significant risk factor for later overweight and obesity [6-8]. Notably, PRWG occurring before the age of one year is associated with a higher risk than PRWG occurring from birth to two years of age [7]. Our previous study in Japan demonstrated that a rise in weight of more than 0.67 standard deviation scores (SDs) between birth and three to four months of age is associated with overweight at three years of age [9]. However, the prevalence of PRWG in Japanese infants up to 1.5 months of age has not yet been evaluated using the WHO z-score or SDs derived from national reference in any published studies.

The objectives of the present study are (1) to convert the national percentile references to WHO WFA z-scores, (2) to assess whether WHO z-scores at birth remain low by 2023 for both sexes, (3) to examine trends in these weight growth patterns up to 2023, and (4) to estimate the prevalence of PRWG up to 11.5 months of age using the WHOCGS and to evaluate whether this prevalence of PRWG differs between WHO WFA z-scores and SDs based on the national weight references.

## Materials and methods

The database used in this study included percentiles, means, and SDs for weight, based on the national growth surveys of preschool children carried out in 2000 and 2010 by the MHLW and in 2023 by the CFA [2-4]. The final participants in the community-based and cross-sectional national surveys comprised 14,115 infants and preschool children aged 14 days to six years at the time of measurement in 2000, 12,426 in 2010, and 11,194 in 2023 [2-4]. Each sample number and percentage from birth to 11.5 months were not available in the reports of the national growth survey on preschool children by the MHLW (2000 and 2010), the CFA (2023), or any other publicly available sources. In the 2000 dataset, records with abnormal measurements were re-examined, and outlier values were excluded. In the second dataset, the percentile and mean values showed random statistical variation due to the limited sample size, so they were smoothed and adjusted using the Tango method [10]. For the 2010 and 2023 percentile data, outliers corresponding to the upper and lower 0.01% of the distribution were excluded, and the data were smoothed and corrected using the LMS method [11]. Importantly, no non-anonymized data of individual Japanese infants and preschool children were used in this study. As this study relied solely on publicly available, open-access databases from the national growth surveys provided by the MHLW and CFA, ethical approval was not required.

Z-scores for WFA based on the WHO reference were initially calculated using an automatic calculator available on the MedCentral website (WHO infant WFA percentiles, 0-24 months) [12]. However, values calculated after one and a half months of age were consistently higher than those reported in a previous study, although the values at birth and one month of age were identical [5]. To validate these discrepancies, a manual calculation was performed using a scientific calculator (Casio fx-570W, Casio Computer Co., Ltd., Japan). The calculation followed the standard WHO formula: z-score = ((weight/M) ^L^-1) / (L x S) and the sex- and age-specific LMS values of WHOCGS were used for this calculation [1]. As a result, the WFA z-scores obtained using both the automatic and manual calculators were identical across all months. The reason for the discrepancy between the WFA z-scores based on WHOCGS in a previous study using the WHO’s igrowup macro and those in this study remains unclear.

“One month” is defined as a 30-day period following birth. Subsequent months are defined as the period from the beginning of each specified month to the end of the following month and are referred to as “the specified month plus half a month”. The change in weight z-score was defined as the difference between the z-score at each monthly age and that at birth. Positive PRWG was defined as an increase of >0.67 in the sex-specific WFA z-score within the first 11.5 months of life [13]. The SDs values based on the national references were calculated using the age- and sex- specific means and standard deviations. Differences of variables in body weight (kg), WFA z-scores, and changes in WFA z-scores among three independent groups in the year 2000, 2010, and 2023 were analyzed separately for each sex using the Kruskal-Wallis test. Statistical analyses were conducted using STATA version 11.0 (Stata Corporation, College Station, TX) and Statview (version 5.0), and a p-value of < 0.05 was considered statistically significant.

## Results

This study yielded four main findings. First, the national percentile data in 2000, 2010, and 2023 were successfully converted to the WHO WFA z-scores using two independent calculators. Second, WHO WFA z-scores, based on relatively low birth weights in kilograms, remained consistent across all percentiles of the Japanese national growth surveys of infants for both sexes in 2000, 2010, and 2023. This stability was observed despite governmental efforts by the MHLW to improve maternal health and nutrition during that period. Specifically, the 50th percentile WFA z-scores for boys were -0.73, -0.73, and -0.60, and for girls -0.63, -0.66, and -0.60, respectively (Tables 1-2).

**Table 1:** The WHO weight-for-age z-scores and postnatal rapid weight gain for the 50th percentile of the Japanese reference for boys All values are rounded to two decimal places. The change in z-score is defined as the difference between the weight z-score at each month and that at birth. “One month” is defined as a 30-day period following birth. Subsequent months are defined as the period from the beginning of each specified month to the end of the following month, and are referred to as “the specified month plus half a month”. Each sample number and percentage from birth to 11.5 months were not available in the reports of the national growth survey on preschool children by the MHLW (2000 and 2010), the CFA (2023), or any other publicly available sources. Differences among groups of boys (a, b, c) over the past three years were analyzed using the Kruskal-Wallis test: a: p>0.5, b: p>0.1, c: p=0.03. The p-value below 0.05 is considered significant. Total*: mean ± standard error, BW: body weight, PRWG: postnatal rapid weight gain, NA: not applicable, mo: month, PAWG >0.67 z-score (±): indicates presence (+) or absence (-) of postnatal rapid weight gain

Year		2000			2010			2023	
Variables	BW (kg)^a^	z-score^b^	Change in z-score^c^ PRWG	BW (kg)^a^	z-score^b^	Change in z-score^c^ PRWG	BW (kg)^a^	z-score^b^	Change in z-score^c^ PRWG
Birth	3.00	-0.73	NA	3.00	-0.73	NA	3.06	-0.60	NA
1.0 mo	4.24	-0.39	0.34 (-)	4.13	-0.59	0.14 (-)	4.15	-0.55	0.05 (-)
1.5 mo	4.90	0.69	1.42 (+)	4.79	0.52	1.25 (+)	4.80	0.54	1.14 (+)
2.5 mo	5.97	0.57	1.30 (+)	5.84	0.39	1.12 (+)	5.80	0.33	0.93 (+)
3.5 mo	6.78	0.53	1.26 (+)	6.63	0.33	1.06 (+)	6.55	0.23	0.83 (+)
4.5 mo	7.35	0.43	1.16 (+)	7.22	0.27	1.00 (+)	7.12	0.15	0.75 (+)
5.5 mo	7.79	0.33	1.06 (+)	7.66	0.18	0.91 (+)	7.56	0.06	0.66 (-)
6.5 mo	8.16	0.26	0.99 (+)	8.00	0.08	0.81 (+)	7.91	-0.03	0.57 (-)
7.5 mo	8.45	0.17	0.90 (+)	8.27	-0.03	0.70 (+)	8.19	-0.12	0.48 (-)
8.5 mo	8.70	0.09	0.82 (+)	8.50	-0.12	0.61 (-)	8.42	-0.21	0.39 (-)
9.5 mo	8.93	0.03	0.76 (+)	8.70	-0.21	0.52 (-)	8.63	-0.28	0.32 (-)
10.5 mo	9.13	-0.04	0.69 (+)	8.88	-0.29	0.44 (-)	8.82	-0.35	0.25 (-)
11.5 mo	9.33	-0.08	0.65 (-)	9.06	-0.35	0.38 (-)	9.00	-0.41	0.19 (-)
Total^*^	7.13±0.6	0.14±0.1	0.95±0.1	7.0±0.6	-0.04±0.1	0.75±0.1	6.92±0.5	-0.1±0.1	0.56±0.1

**Table 2:** The WHO weight-for-age z-scores and postnatal rapid weight gain for the 50th percentile of the Japanese national reference for girls Differences among groups of girls (d, e, f) over the past three years were analyzed using the Kruskal-Wallis test: d, e, and f: p>0.5. The p-value below 0.05 is considered significant.

Year		2000			2010			2023	
Variables	BW (kg)^d^	z-score^e^	Change in z-score^f^ PRWG	BW (kg)^d^	z-score^e^	Change in z-score^f^ PRWG	BW (kg)^d^	z-score^e^	Change in z-score^f^ PRWG
Birth	2.95	-0.63	NA	2.94	-0.66	NA	2.95	-0.63	NA
1.0 mo	4.01	-0.31	0.32 (-)	3.89	-0.53	0.13 (-)	3.91	-0.50	0.13 (-)
1.5 mo	4.64	0.76	1.39 (+)	4.47	0.48	1.14 (+)	4.44	0.43	1.06 (+)
2.5 mo	5.57	0.64	1.27 (+)	5.42	0.43	1.09 (+)	5.33	0.30	0.93 (+)
3.5 mo	6.24	0.52	1.15 (+)	6.15	0.40	1.06 (+)	6.06	0.29	0.92 (+)
4.5 mo	6.75	0.40	1.03 (+)	6.71	0.35	1.01 (+)	6.64	0.27	0.90 (+)
5.5 mo	7.18	0.33	0.96 (+)	7.14	0.28	0.94 (+)	7.10	0.23	0.86 (+)
6.5 mo	7.54	0.27	0.90 (+)	7.47	0.19	0.85 (+)	7.47	0.19	0.82 (+)
7.5 mo	7.82	0.19	0.82 (+)	7.75	0.12	0.78 (+)	7.75	0.12	0.75 (+)
8.5 mo	8.05	0.10	0.73 (+)	7.97	0.02	0.68 (+)	7.99	0.04	0.67 (-)
9.5 mo	8.26	0.03	0.66 (-)	8.17	-0.06	0.60 (-)	8.19	-0.04	0.59 (-)
10.5 mo	8.46	-0.02	0.61 (-)	8.34	-0.14	0.52 (-)	8.36	-0.12	0.51 (-)
11.5 mo	8.67	-0.05	0.58 (-)	8.51	-0.20	0.46 (-)	8.53	-0.18	0.45 (-)
Total^*^	6.63±0.5	0.17±0.1	0.87±0.1	6.53±0.5	0.05±0.1	0.77±0.1	6.52±0.5	0.03±0.1	0.72±0.1

**Figure 1:**
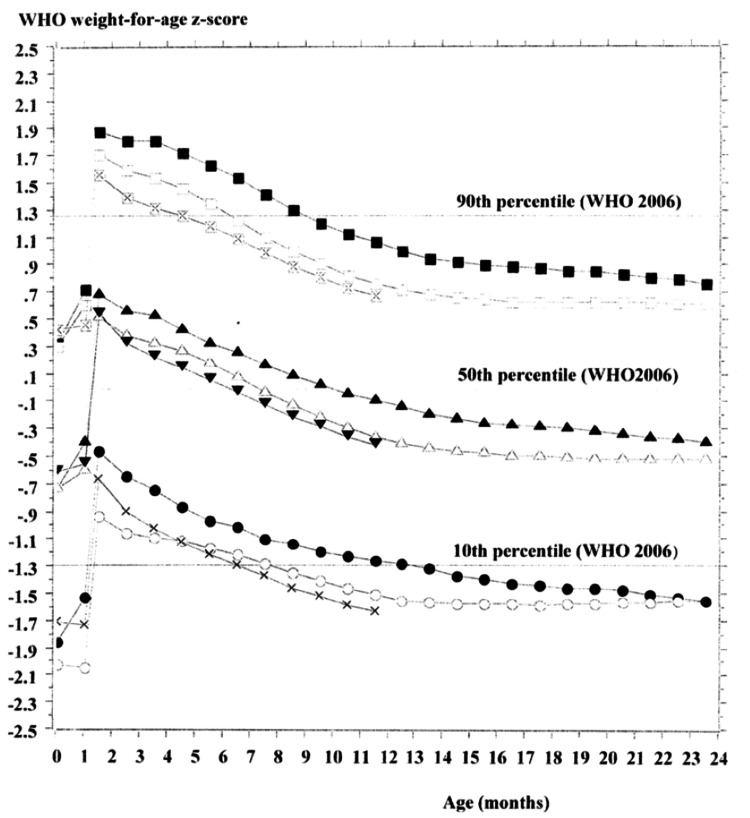
Conversion for each weight percentile from the Japanese national references in 2000, 2010, and 2023 to sex-specific weight-for-age z-scores based on the WHO growth standards for boys ●: 2000 10th percentile, ○: 2010 10th percentile, ×: 2023 10th percentile ▲: 2000 50th percentile, △: 2010 50th percentile, ▼: 2023 50th percentile ■: 2000 90th percentile, □: 2010 90th percentile, : 2023 90th percentile

**Figure 2:**
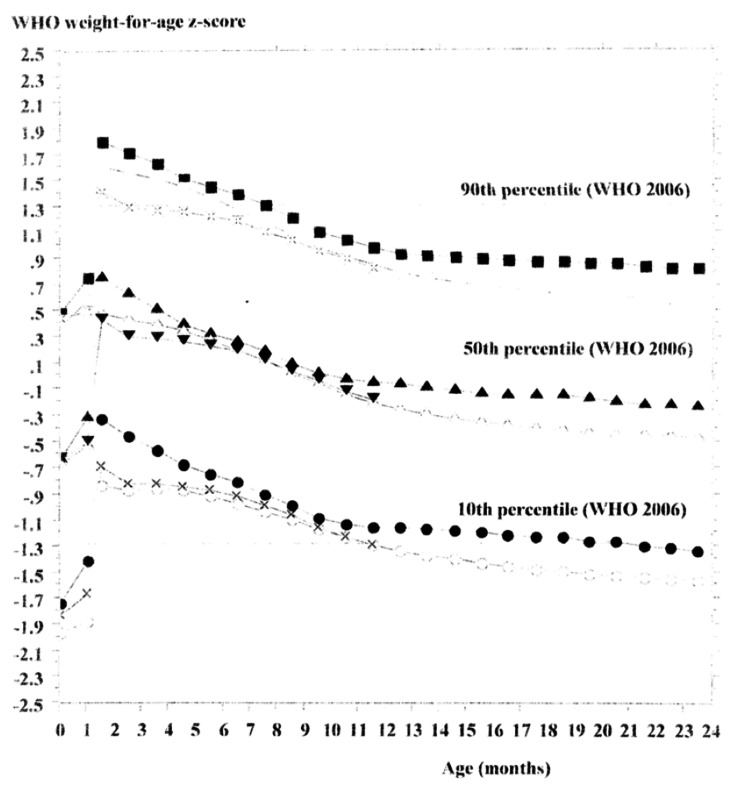
Conversion for each weight percentile from the Japanese national references in 2000, 2010, and 2023 to sex-specific weight-for-age z-scores based on the WHO growth standards for girls

Third, WFA z-scores peaked at 1.5 months of age in both sexes and subsequently declined across all percentiles relative to the WHOCGS - with the exception of the 10th percentile among girls in 2000. Furthermore, for both boys and girls, WFA z-score trajectories were the highest in 2000 and showed a steady decline by 2023. These trends suggest a divergence between the growth patterns of Japanese infants and those represented in the WHOCGS (Figures 1-2).

Fourth, while the 50th percentile line of the national reference statistically represents the median, not all infants born at this percentile consistently follow the same trajectory up to 11.5 months of age. If infants were to maintain their birth percentile throughout infancy, WFA z-scores would peak at 1.5 months and then decline gradually. Among the three independent groups from 2000, 2010, and 2023, differences in the continuous variable of change in WFA weight z-scores were observed only in boys in 2023 (p=0.0303). Additionally, PRWG persisted until 10.5 months of age in boys and 8.5 months of age in girls in 2000 (Tables 1-2). As shown in Tables 1-2, the prevalence of PRWG appears to be overestimated when using WFA z-scores derived from the WHOCGS during early infancy, particularly for both sexes in the year 2000, relative to the national references. Changes in SDs also indicated the presence of PRWG at 2.5 months in girls, which then diminished gradually and was no longer evident by 11.5 months of age (Table 3).

**Table 3:** Postnatal rapid weight gain derived from mean and standard deviation based on the 50th percentile of the Japanese national reference in 2000 All values are rounded to two decimal places. Mean and SD values are taken from the 2000 national reference. The SD score is calculated as (median body weight – mean) /SD. The change in SD score is defined as the SD score at each month minus that at birth. mo: month, SDs: standard deviation scores, NA: not applicable, PRWG: postnatal rapid weight gain > 0.67 SDs (presence or absence) based on the national reference

	Boys			Girls		
	SD score	Change in SD score	PRWG	SD score	Change in SD score	PRWG
Birth	0	NA	NA	-0.13	NA	NA
1.5 mo	-0.1	-0.10	-	-0.18	-0.05	-
2.5 mo	0.67	0.67	-	0.62	0.75	+
3.5 mo	0.48	0.48	-	0.34	0.47	-
4.5 mo	0.28	0.28	-	0.19	0.32	-
5.5 mo	0.11	0.11	-	0.23	0.36	-
6.5 mo	0.18	0.18	-	0.05	0.18	-
7.5 mo	0.28	0.28	-	0.03	0.16	-
8.5 mo	0.10	0.10	-	0.06	0.19	-
9.5 mo	0.03	0.03	-	0.07	0.20	-
10.5 mo	0.03	0.03	-	-0.04	0.09	-
11.5 mo	0.14	0.14	-	0.08	0.21	-

## Discussion

The decrease in mean birth weight (kg) and the increase in the prevalence of low birth weight neonates (<2,500 g) observed in Japan since 1980 are striking when compared to trends in other developed countries [14]. Even when the national reference for the 50th weight percentile is converted to WHO z-scores, birth weights for both sexes have remained -0.6 or less up to 2023. Several potential factors have been proposed to explain this trend, including multiple gestation, shorter gestational age, fetal sex, birth order, paternal age, lower pre-pregnancy body mass index (BMI), and dietary restrictions among young women [15]. A large-scale Japanese cohort study also reported that insufficient weight gain during pregnancy - particularly from the second trimester onwards - is associated with an increased prevalence of low birth weight neonates, regardless of pre-pregnancy BMI [16].

The WHO WFA z-score curves, derived from Japanese national percentiles up to 2023, display characteristics that differ from the percentile lines of the WHOCGS. While these differences may suggest a discrepancy in infant growth patterns, it is important to note that the WHOCGS is based on singleton, full-term, and exclusively breastfed infants [1]. In contrast, the national reference data from 2000 included infants who were breastfed, formula-fed, or received mixed feeding and reported that 10.7% of neonates were born with low birth weight [2].

The mean WHO WFA z-score in European infants decreases from birth until approximately one to two months of age, after which it gradually increases [17,18]. In contrast, among Japanese infants, the 50th percentile WFA curve for both sexes based on WHO z-scores shows a notable increase until around 1.5 months of age, followed by a gradual decline up to 23.5 months. Furthermore, the Japanese weight SD scores peaked slightly later, at 2.5 months of age, and subsequently showed a decreasing trend until 11.5 months, similar to the WHO weight z-scores.

Tables 1-2 suggest a potential overestimation of the prevalence of PRWG, particularly for both sexes in the year 2000, when using the WFA z-scores based on the WHOCGS during early infancy, compared to the national references. Conversely, the prevalence of PRWG, when assessed via postnatal change in SDs, appears to be markedly lower than that using WHO z-scores (Table 3). In a previous retrospective study, we found that the prevalence of PRWG up to three to four months of age in 1,351 healthy infants was 22.7%, as determined using SDs from the national reference [9]. A review of the prevalence of PRWG up to two years of age has reported a range from 10% to 40%, with an exception of 54.2% in 203 infants aged ≤4 months in a low-income, inner-city minority population in the United States [7,19]. These findings suggest that it may be more appropriate to assess PRWG in early infancy using SDs derived from national reference data rather than WHO z-scores.

SDs values remained above zero from 2.5 to 11.5 months of age, except for the value of 10.5 months of age in girls, indicating that the median weights of Japanese infants were higher than the average values used in the reference. These results follow from a negatively skewed distribution. Notably, postnatal changes in SDs peaked at 2.5 months of age in both sexes, and PRWG in girls was observed exclusively at this time point. This may reflect the pronounced negative skewness in the distribution (Table 3).

A comparison of the prevalence of PRWG from birth to three months of age between the 2008 CDC reference and the 2006 WHOCGS showed that the prevalence based on the CDC reference was 14.3% higher than that based on the WHOCGS [20]. Aside from this report, no other studies comparing the prevalence of PRWG from birth to 1.5 months or three months of age between the national references and the WHOCGS have been identified. Further investigation is needed to determine the extent to which PRWG, derived from the WHO weight z-score of Japanese infants up to 1.5 months of age, represents a risk for subsequent overweight or obesity, and the extent to which catch-up growth due to intrauterine growth retardation in low birth weight infants (<2,500 g) contributes to PRWG.

PRWG of full-term infants with normal weight at birth may be due, in part, to over-nutrition during early infancy. The national surveys on Japanese infant nutrition until 2023 were a simple questionnaire that focused on nutritional modes, such as breastfeeding, formula feeding, and mixed feeding. In 2000, the proportions of infant feeding methods at 1.5 months of age were as follows: 44.8% breastfed, 11.2% formula-fed, and 44.0% mixed-fed [2]. These results showed a lack of accurate data on calorie and protein intakes during early infancy. Therefore, there is no direct evidence that over-nutrition or over-feeding, as indicated by the WHO WFA z-scores within the first 1.5 months of life, causes PRWG.

There were three limitations in this study. First, it was not possible to clarify how the different prevalence of PRWG based on WHOCGS or SDs derived from the national references from birth to early infancy contributes to overweight and/or obesity risk later in life. Second, the reason why the WHO weight z-scores reported by Kobayashi et al. [5] after 1.5 months of age were different from those in this study could not be elucidated. Third, no information on energy or protein intake during the first 1.5 months of life was available from the national nutrition survey or other sources.

## Conclusions

Weight growth patterns based on WHO z-scores revealed a steady downward trend through 2023. The prevalence of PRWG may be overestimated because of the higher weight values observed after 1.5 months of age and the lower birth weight values, as reflected in WHO z-scores converted from the Japanese national percentile references for 2000, 2010, and 2023. Therefore, SDs derived from national references may currently provide a more appropriate basis for assessing the prevalence of PRWG during early infancy than that based on the WHO z-score. Furthermore, caution should be taken when comparing results from studies employing different evaluation criteria of PRWG.
